# MASCC multidisciplinary evidence-based recommendations for the management of malignant bowel obstruction in advanced cancer

**DOI:** 10.1007/s00520-022-06889-8

**Published:** 2022-03-10

**Authors:** Ainhoa Madariaga, Jenny Lau, Arunangshu Ghoshal, Tomasz Dzierżanowski, Philip Larkin, Jacek Sobocki, Andrew Dickman, Kate Furness, Rouhi Fazelzad, Gregory B Crawford, Stephanie Lheureux

**Affiliations:** 1 Department of Medical Oncology, Princess Margaret Cancer Centre, Toronto, Canada; 2 Autonomous University of Barcelona, Barcelona, Spain; 3 12 Octubre University Hospital, Madrid, Spain; 4 Department of Supportive Care, Princess Margaret Cancer Centre, Toronto, Canada; 5 Laboratory of Palliative Medicine, Department of Social Medicine and Public Health, Medical University of Warsaw, Warsaw, Poland; 6 Palliative and Supportive Care Service, Lausanne University Hospital and University of Lausanne, Lausanne, Switzerland; 7 Department of General Surgery and Clinical Nutrition, Centre for Postgraduate Medical Education, Warsaw, Poland; 8 Academic Palliative and End of Life Care Department, Royal Liverpool and Broadgreen University Hospitals NHS Trust, Liverpool, England UK; 9 Department of Dietetics, School of Health Sciences, Swinburne University of Technology, Hawthorn, Victoria Australia; 10 Library and information services, University of Health Network, Toronto, Canada; 11 Northern Adelaide Palliative Service, Northern Adelaide Local Health Network, Adelaide, Australia; 12 Discipline of Medicine, University of Adelaide, Adelaide, Australia

**Keywords:** Malignant bowel obstruction, Palliative Care, Cancer—gastrointestinal, Gynecologic neoplasm, Guidelines

## Abstract

**Purpose:**

To provide evidence-based recommendations on the management of malignant bowel obstruction (MBO) for patients with advanced cancer.

**Methods:**

The Multinational Association for Supportive Care in Cancer (MASCC) MBO study group conducted a systematic review of databases (inception to March 2021) to identify studies about patients with advanced cancer and MBO that reported on the following outcomes: symptom management, bowel obstruction resolution, prognosis, overall survival, and quality of life. The review was restricted to studies published in English, but no restrictions were placed on publication year, country, and study type. As per the MASCC Guidelines Policy, the findings were synthesized to determine the levels of evidence to support each MBO intervention and, ultimately, the graded recommendations and suggestions.

**Results:**

The systematic review identified 17,656 published studies and 397 selected for the guidelines. The MASCC study group developed a total of 25 evidence-based suggestions and recommendations about the management of MBO-related nausea and vomiting, bowel movements, pain, inflammation, bowel decompression, and nutrition. Expert consensus-based guidance about advanced care planning and psychosocial support is also provided.

**Conclusion:**

This MASCC Guideline provides comprehensive, evidence-based recommendations about MBO management for patients with advanced cancer.

**Supplementary Information:**

The online version contains supplementary material available at 10.1007/s00520-022-06889-8.

## Introduction

MBO is a severe complication in advanced cancer [1]. MBO was defined in the International Conference on MBO and Clinical Protocol Committee as (i) clinical evidence of bowel obstruction (via history, physical, and/or radiological examination), (ii) bowel obstruction beyond the ligament of Treitz, and (iii) diagnosis of intra-abdominal cancer with an incurable disease, or a non-intra-abdominal primary cancer with the clear intraperitoneal disease [1]. The algorithm of care for patients with MBO is not uniform and often differs according to clinical factors (e.g., prognosis) and between institutions and countries.

In this article, the MASCC MBO multidisciplinary study group presents comprehensive, evidence-based recommendations about MBO management for patients with advanced cancer and the methodology used to develop these recommendations.

## Background

### Etiology

MBO most frequently occurs when patients have advanced cancers that originate in the abdomen or pelvis. Though the incidence of MBO is not well established, based on retrospective and autopsy-based studies, MBO is estimated to occur in 10–28% of patients with gastrointestinal cancers and up to 51% of patients with advanced ovarian cancer [2]. Limitations of these estimates include patient population selection and nonhomogeneous criteria to diagnose MBO.

The mechanisms of MBO development are multi-factorial and can be divided into two main groups: mechanical and functional obstruction [2, 3]. Causes of mechanical obstruction include extrinsic obstruction of the lumen by pathology, such as mesenteric and omental masses, adhesions, and fibrosis; intra-luminal obstruction from tumor growth in the bowel; and intra-mural obstruction by tumor within the bowel wall, which impairs motility. Whereas functional obstruction is a result of motility disorders, which can be due to tumor infiltration of mesentery, nerves and/or celiac and enteric plexus, and paraneoplastic syndromes. Further, nonmalignant factors may induce or worsen bowel obstruction (BO) in patients with advanced cancer, including constipation/fecal impaction, pharmacological (i.e., opioids, intra-peritoneal chemotherapy), fibrosis, and adhesions from prior surgery and radiotherapy.

### Pathophysiology and symptoms

MBO can occur in the small or large bowel, with small BO being more common [3, 4]. The obstruction can be partial or complete and can occur at single or multiple transition points. MBO causes reduction or absence of movements of the intestinal content and bowel distension [3, 4]. The accumulation of content in the intestinal lumen increases the epithelial surface area and prompts an accumulation of gastric, pancreatic, biliary secretions, water, and salt, which damages the intestinal epithelium and triggers an inflammatory response with intestinal edema, hyperemia, and production of inflammatory mediators (i.e., prostaglandins, vasoactive intestinal polypeptide, and nociceptive mediators). Bacterial overgrowth and translocation are important mechanisms in the development of symptoms. The cumulative impact of these events results in abdominal pain, cramps, distention, nausea, vomiting, absence of gas and stool passage, and, occasionally, overflow diarrhea. MBO symptoms usually start gradually and become more frequent and severe when a complete obstruction occurs. Re-obstruction and malnutrition are common, with malnutrition being an independent predictor of poor survival in this population [5–8].

### Diagnosis

MBO is a clinical diagnosis that is confirmed with radiological imaging. Historically, abdominal x-rays were recommended to be the initial imaging modality, but their sensitivity to detect MBO is moderate and poses challenges in detecting the exact site, cause, or complications derived from MBO [9]. Contrast computed tomography (CT) is more valuable as it provides diagnostic precision [4]. The American College of Radiology recommends the use of CT of the abdomen and pelvis with intravenous (IV) contrast for patients with suspected acute small BO and either a CT abdomen and pelvis with IV contrast or a CT enterography for patients suspected to have intermittent or low-grade small BO [9].

### Advanced care planning and goals of care conversations

When patients with advanced cancers are diagnosed with MBO, clinicians should acknowledge that medical decisions are directed at extending life, minimizing distressing symptoms, and maximizing quality of life. Care should be holistic and person-centered with focus on the interrelationship between physical, psychosocial, and spiritual issues [10]. Clinicians should encourage patients to substitute decision-makers with who they can communicate their values and goals to assist them with medical decision-making when they no longer have the capacity. In some jurisdictions, written advance care documents are legislated and may include options of appointing a formal decision-maker on the loss of capacity, statements about values and goals, and even binding refusals of specific interventions.

Before offering interventions for MBO management, clinicians should take into consideration the risks associated with the interventions in addition to the patient’s estimated prognosis, performance status, comorbidities, location of care and ease of further assessment and support (e.g., home parenteral nutrition programs may not be universally available). Financial implications may also impact whether an intervention might be offered or undertaken. Furthermore, patients may have strongly held views about the interventions that they would accept and their decisions can change over time, especially when the benefit of therapy or intervention may no longer exceed the burden, and cessation may be considered appropriate by their clinicians or even desired by the patient. Clinicians are encouraged to discuss these possible scenarios with patients and their families before commencing interventions, such as total parenteral nutrition. As patients approach the end of their lives, their goals of care may shift from disease management and life prolongation to symptom management and quality of life [11].

The interventions that might be offered or considered clinically appropriate may depend on the estimated prognosis of the patient. Physician prediction of survival is known to be inaccurate and often overly optimistic. Objective factors associated with a short prognosis include deteriorating performance status and the presence of other symptoms, potentially indicative of progressive malignant disease in other organs [12]. Laboratory findings associated with systemic inflammatory response (e.g., elevated C-reactive protein), reduced albumin, and leucocytosis are also associated with poor prognosis [13]. Prognostic models incorporating physician prediction of survival and clinical and laboratory factors improve the accuracy of clinical prediction [14].

The treating clinician should offer sensitive discussions with patients and their families about different treatment options that may be possible, what outcomes might be reasonably expected, possible adverse effects or burdens, and how to measure whether such interventions may provide some success in terms of longevity, symptom control or improvement in quality of life. Assessments of a patient’s illness understanding and decision-making wishes around disclosure and acknowledging emotional responses are essential [15]. Exploring these issues requires sensitivity and excellent communication skills. Generally, personal autonomy and the right to be involved in all medical decision-making is a widely held, but not a universal, principle. Some patients may choose not to confront their own mortality or lack the capacity to do so. In certain cultures, patients may choose to defer medical decision-making to their family. Goals of care conversations require careful exploration, always with the patient’s best interest being foremost.

## Methods

### Literature review

An information specialist (RF) conducted a systematic literature search in Medline ALL (Medline and Epub Ahead of Print and In-Process, In-Data-Review, and Other Non-Indexed Citations), Embase Classic +Embase, Cochrane Central Register of Controlled Trial, Cochrane Database of Systematic Reviews, PsycInfo, all from the OvidSP platform; Scopus from Elsevier, and Global Index Medicus (LILACS, WPRIM, IMEMR, IMSEAR, and AIM) from WHO. The literature search was executed from the inception of each database to March 2021 with no language limitations. Each search strategy comprised a combination of controlled vocabulary terms and text words, adapting the database-specific search syntax. Where applicable, the search was restricted to human studies, adults ≥18 years of age and excluded books, conference abstracts, and dissertations. Appendix A presents the Medline ALL search.

**Fig. 1:**
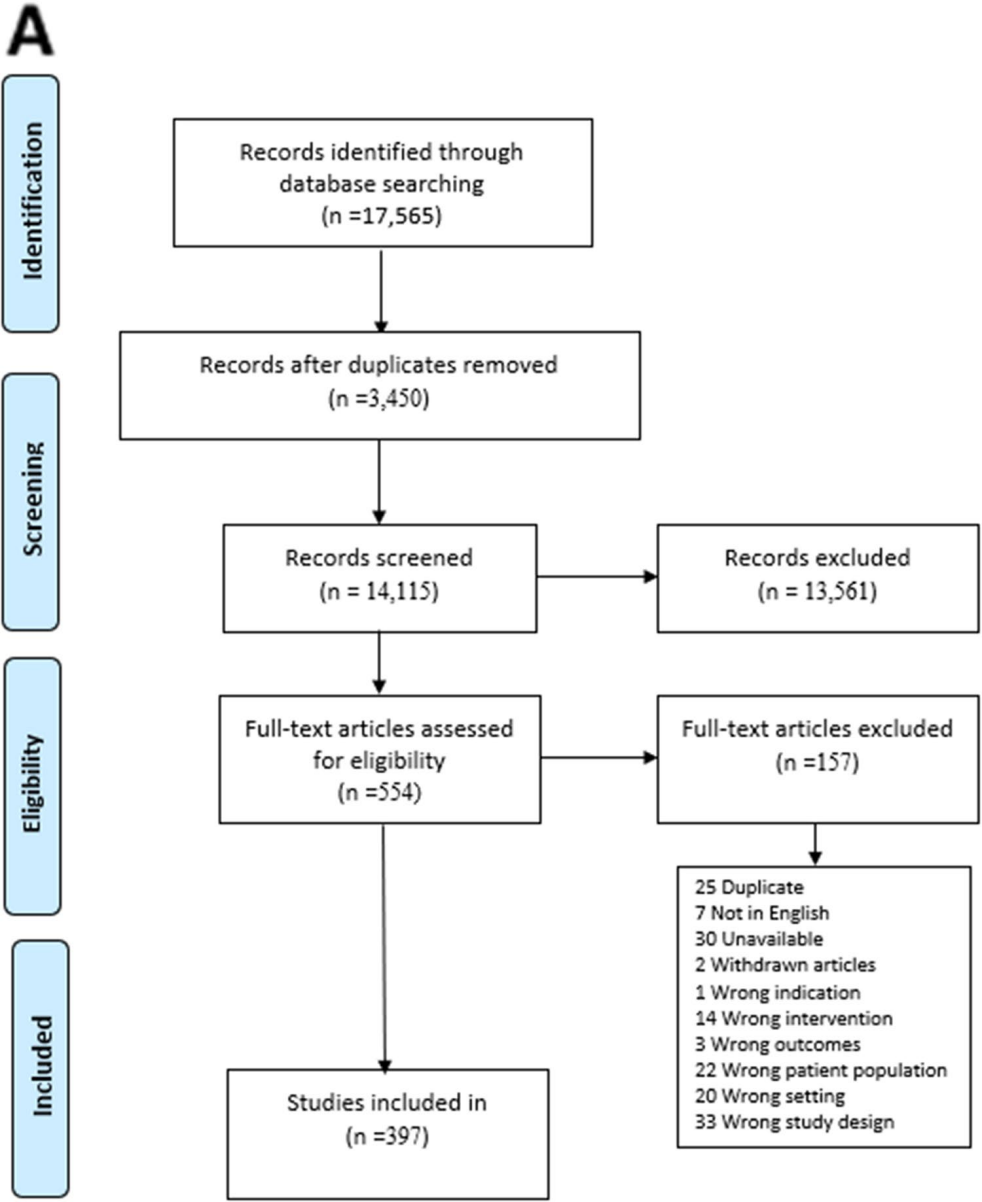

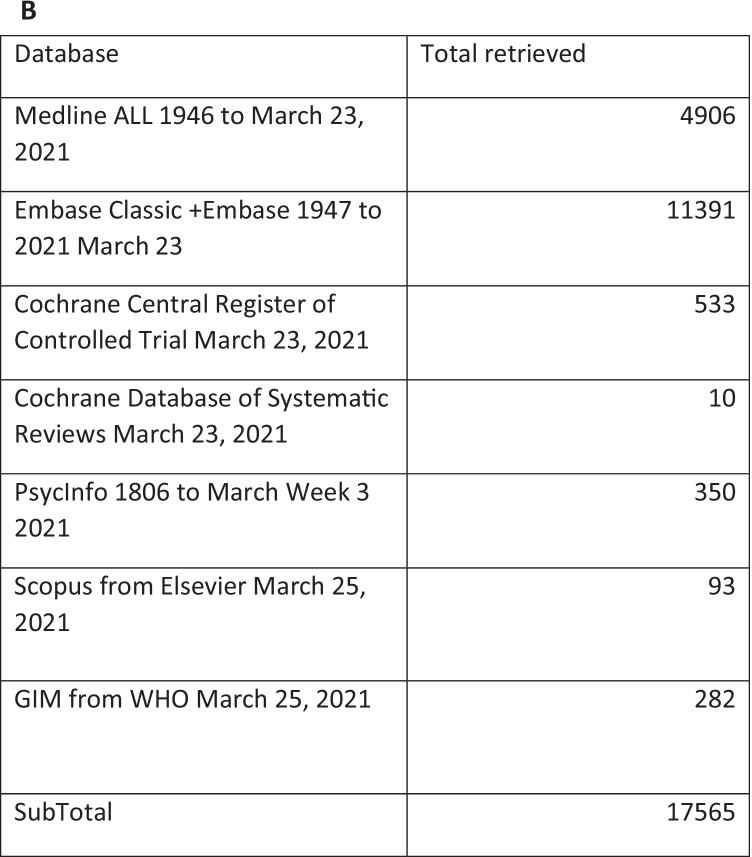
PRISMA flow diagram (**A**) and chart numbers (**B**)

Records obtained were stored on EndNote citation software, following which duplicates were removed and studies uploaded onto Covidence. Abstracts were screened for eligibility, and then full-text articles were assessed. Any discrepancies in study selection were resolved through consensus. Inclusion criteria were studies about people with advanced cancers (any type) with MBO (small and/or large bowel) that examined MBO interventions and reported on outcomes related to symptom management, BO resolution, prognosis, overall survival, and/or quality of life. All types of primary research studies (with or without comparators) were included. Studies about anti-cancer treatments (e.g., chemotherapy, radiation) for MBO management and non-English studies were excluded. In total, 17,565 studies were identified, and 3450 duplicates removed. From 14,115 studies that were screened against title and abstract, 13,561 were excluded. A total of 554 studies were assessed for full-text eligibility, and 397 studies were included for this review. Figure 1 presents the PRISMA flow diagram. Using the 2018 *MASCC Guidelines Policy: recommendations for MASCC guideline construction and the endorsement of externally generated guidelines*, the level of evidence (Table 1) for each MBO intervention was determined and synthesized into suggestions or recommendations with gradings (Table 2). “Suggestions” are used for statements that are based on level III, IV, or level V evidence. “Recommendations” are reserved for statements that are based on level I or level II evidence. Whereas “no guideline possible” are used when there is insufficient evidence on which to base a guideline. This implies that there is little or no evidence regarding the practice in question or that the panel lacks consensus on the interpretation of existing evidence.

**Table 1:** MASCC levels of evidence

Level	Criteria
I	Evidence obtained from meta-analysis of multiple, well-designed, controlled studies; randomized trials with low false-positive and false-negative errors (high power).
II	Evidence obtained from at least one-well designed experimental study; randomized trials with high false-positive and/or false-negative errors (low power).
III	Evidence was obtained from well-designed, quasi-experimental studies, such as nonrandomized, controlled single-group, pretest-posttest comparison, cohort, time, or matched case-control series.
IV	Evidence was obtained from well-designed, non-experimental studies, such as comparative and correlational descriptive and case studies.
V	Evidence obtained from case reports and clinical examples.

Adapted from MASCC (2018). *MASCC Guidelines Policy: Recommendations for MASCC Guideline Construction and the Endorsement of Externally Generated Guidelines.* https://www.mascc.org/assets/Toolbox/PoliciesForms/mascc_guideline_policy_2018.pdf

**Table 2:** MASCC grading of recommendations

Grade	Evidence needed
A	Evidence of type I or consistent findings from multiple studies of type II, III, or IV.
B	Evidence of types II, III, or IV and findings are generally consistent.
C	Evidence of types II, III, or IV and findings are inconsistent.
D	Little or no systematic empirical evidence.

Adapted from MASCC (2018). *MASCC Guidelines Policy: Recommendations for MASCC Guideline Construction and the Endorsement of Externally Generated Guidelines.* https://www.mascc.org/assets/Toolbox/PoliciesForms/mascc_guideline_policy_2018.pdf

## Suggestions/recommendations

The following sections present each MBO management recommendation and its associated evidence (Table 3). In complete MBO, medication should be administered intravenously or subcutaneously if available.

**Table 3:** Summary of suggestions and recommendations for MBO management, with associated level and grade of evidence

**Interventions**	**Level Evidence**	**Grade**
Anti-emetics		
The benefit of anticholinergics (hyoscine butylbromide) may be inferior to octreotide to reduce vomiting in MBO.	III	D
Haloperidol, a butyrophenone antipsychotic, may be an effective anti-emetic in MBO, particularly for complete MBO.	IV	B
Dopamine antagonist prokinetic drugs (e.g., metoclopramide, domperidone) may be effective for the management of nausea, vomiting and restoring bowel transit time in partial MBO. Due to the potential increased risk of bowel perforation, it likely should be avoided in complete MBO.	III	B
Histamine H_1_ antagonists, (e.g., dimenhydrinate, cyclizine) may be an effective anti-emetic in complete MBO.	IV	D
Phenothiazines (e.g., chlorpromazine) may reduce nausea and vomiting in MBO.	IV	D
Granisetron, serotonin (5HT_3_) antagonist may reduce nausea and the frequency of vomiting in MBO.	III	D
Somatostatin analog (octreotide, lanreotide) may reduce vomiting in MBO	I	A
Thienobenzodiazepene antipsychotic (e.g., olanzapine) may reduce nausea and vomiting in MBO.	IV	D
Laxatives		
Oral osmotic laxatives should be considered in the management of impaired bowel movements in partial bowel obstruction but should be avoided in complete MBO.	V	D
Analgesics		
Opioids are commonly used to treat pain associated with MBO, but there is no evidence to support their use.	V	D
The benefit of anticholinergics (hyoscine butylbromide) may be effective to reduce abdominal pain in MBO.	III	D
Corticosteroids		
The use of steroids may help with the acute symptoms of MBO and can be used for short-term benefits.	III	B
Bowel decompression		
Nasogastric tube may be used for temporary decompression in acute MBO.	V	D
Endoscopic or percutaneous gastrostomy tube may be used for gastric decompression in MBO.	IV	B
Percutaneous transesophageal gastro-tubing may be used for gastric decompression in MBO.	IV	C
Palliative surgery and stent		
Self-expanding metallic stents are the preferred alternative for the management of single-level large bowel obstruction when technically feasible and in the absence of colonic perforation.	II	B
In the case of a multi-level obstruction, palliative surgical intervention may be considered in a highly selected population.	IV	B
Patients with advanced cancer that undergo palliative surgery for MBO are at high risk of surgical complications, and less invasive surgical interventions should be considered.	IV	B
Nutrition		
When a patient is initially diagnosed with MBO, they should be made Nil Per Os (nothing by mouth), and then when the acute MBO resolves fully or partially, a symptom led, slow and graded reintroduction to oral diet is recommended. This may include clear fluids, free or full fluids, texture modified low fiber diet (soft, minced, and pureed), and if tolerated back to normal textured low fiber diet.	IV	B
Nutrition interventions should be initiated in patients with advanced cancers only where the benefits of these interventions on quality of life and survival outweigh the risks, with clear expectations discussed by a multidisciplinary team with patients and families.	IV	B
Parenteral hydration does not prevent or improve symptoms, such as thirst or dry mouth, nor does it increase survival, and in excessive amounts, it may bring on fluid overload, peripheral, and pulmonary edema.	III	B
Parenteral hydration should not be initiated routinely in the last days of life.	III	B
Home parenteral nutrition may be beneficial and maintain the quality of life in a very selected group of patients with MBO.	IV	D
Central venous access is preferred for home parenteral nutrition delivery.	III	B
In an end-of-life home, parenteral nutrition should be discontinued (or not initiated) as it raises the risk of complications and may prolong suffering.	V	D

### Anti-emetics

The 2021 MASCC guideline about select pharmacologic management of nausea and vomiting in MBO was released in August 2021 as an update to a previous guideline published in 2017 [16]. The following sections and Appendix B provide suggestions/recommendations for all anti-emetic drug classes reportedly used for MBO management and their associated existing evidence:

#### Anticholinergics

*Suggestion: The benefit of anticholinergics (hyoscine butylbromide) may be inferior to octreotide to reduce vomiting in MBO. (Level of evidence: III; Grade: D).*

Hyoscine (scopolamine) butylbromide is an anticholinergic agent that reduces gastrointestinal secretions. A 2016 systematic review [17] identified four randomized clinical trials with high Cochrane risk of bias that found somatostatin analog (e.g., octreotide) were more effective than hyoscine butylbromide in reducing nausea and vomiting [18–21]. Three case reports reported mixed findings, where two reported hyoscine butylbromide in combination with other drugs (e.g., octreotide, dexamethasone) was effective in reducing vomiting [22, 23], and one that reported that it was ineffective for controlling vomiting [24]. Since the publication of that review, no new randomized clinical trials have been published. An alternative anticholinergic that can reportedly be used is glycopyrrolate. However, this review only identified one case report that reported that glycopyrrolate reduced nausea and vomiting in MBO [25]. Appendix B presents the characteristics of all included studies about anticholinergic use in MBO.

#### Butyrophenone antipsychotic

*Suggestion: Haloperidol, a butyrophenone antipsychotic, may be an effective anti-emetic in MBO, particularly for complete MBO (level of evidence: IV; grade: B),*

Butyrophenone antipsychotics are commonly used as anti-psychotics for delirium management but are also used for the management of nausea and vomiting in MBO. The main butyrophenone used is haloperidol. The review identified two cross-sectional studies [26, 27] and two case reports/series that reported on haloperidol’s use in MBO [25, 28]. All studies suggested that haloperidol effectively relieve nausea and vomiting in MBO and is the preferred anti-emetic in complete BO [25–28].

#### Dopamine antagonist prokinetic

*Suggestion: Dopamine antagonist prokinetic drugs (e.g., metoclopramide, domperidone) may be effective for the management of nausea, vomiting, and restoring bowel transit time in partial MBO. Due to the potential increased risk of bowel perforation, it likely should be avoided in complete MBO. (level of evidence: III; grade B)*

Dopamine antagonist prokinetic drugs block dopamine receptors. They increase lower esophageal sphincter pressure, gastric motility, and, therefore, gastric emptying [29]. The main prokinetic drugs used in MBO management are domperidone and metoclopramide. We identified two cross-sectional studies [26, 27] and three case reports/series [24, 30, 31] published between 1985 and 2016, that reported on their use in MBO. Of these studies, three studies found metoclopramide was effective for nausea, vomiting, and restoring intestinal transit time [27, 30, 31], particularly in incomplete BO [27]. However, two studies found that metoclopramide was ineffective for relieving these symptoms [24, 26]. Only one study reported on the use of domperidone and found it ineffective for control of vomiting [26].

#### ***Histamine (H***_***1***_***) antagonist***

*Suggestion: Histamine H*_*1*_ *antagonists (e.g., dimenhydrinate, cyclizine) may be an effective anti-emetic in complete MBO (level of evidence: IV; grade D).*

Histamine H_1_ antagonists are a drug class that is primarily used for the management of motion sickness from vestibular stimulation. The main histamine H_1_ antagonist that is used is dimenhydrinate. This review identified only one cross-sectional study published in 1994 that reported histamine H_1_ antagonist use for nausea management in complete BO [27].

#### Phenothiazines

*Suggestion: Phenothiazines (e.g., chlorpromazine) may reduce nausea and vomiting in MBO (level of evidence: IV; grade: D).*

Phenothiazines are first-generation antipsychotic agents that can be used for the prevention and control of nausea and vomiting. Examples of phenothiazines are chlorpromazine, prochlorperazine, and methotrimeprazine (also known as levomepromazine). The review identified one cross-sectional study that found chlorpromazine, methotrimeprazine, and prochlorperazine effectively reduce nausea and vomiting in patients with MBO [26].

#### ***Serotonin (5-HT***_***3***_***) antagonists***

*Suggestion: Granisetron, serotonin (5HT*_*3*_ *) antagonist may reduce nausea and the frequency of vomiting in MBO (level of evidence: III; grade D).*

Serotonin (5-HT_3_) antagonists act on receptors located in the chemoreceptor trigger zone to reduce nausea and vomiting. The review identified only one study published in 2009 that examined the use of a 5-HT_3_ antagonist, granisetron, for MBO management [32]. This study was a phase II clinical trial that found granisetron, in addition to dexamethasone and as-needed haloperidol, significantly reduced the severity of nausea (*p* < 0.001) and a number of episodes of vomiting (*p* < 0.001) before and after treatment. However, the reported incidence of constipation associated with granisetron ranges from 3 to 18% [29]. Further studies are required to assess its use in MBO.

#### Somatostatin analog

*Recommendation: Somatostatin analog (octreotide, lanreotide) may reduce vomiting in MBO (levels of evidence: I; grade A).*

MBO can cause intestinal secretions to accumulate and contribute to bowel distention, resulting in nausea and vomiting. Octreotide is a somatostatin analog that reduces intestinal and pancreas secretion and gastrointestinal motility, biliary contraction, and intestinal edema [20, 33]. Octreotide may be administered by subcutaneous bolus or continuous subcutaneous infusion. Its duration of activity is approximately 6 to 12 h, with an average half-life elimination of 1.8 h, thus necessitating multiple daily dosing schedules. Given it has a short half-life, a long-acting depot formulation is available to be administered intramuscularly once a month. Furthermore, another somatostatin analog, lanreotide, is available as a long-acting depot formulation [34, 35].

A 2016 systematic review of randomized control trials and quasi-randomized control trials, published between 1979 and 2016, identified seven studies that compared the effect of somatostatin analog with placebo and/or other pharmacologic agents (e.g., hyoscine butylbromide) on vomiting [17]. A meta-analysis was not possible given the heterogeneity in study design, outcomes, and timing of endpoints [17]. Of these seven studies, five studies with high Cochrane risk of bias found the somatostatin analog were more effective than hyoscine butylbromide [18–21] and placebo [36] for reducing vomiting. Whereas two studies with low Cochrane risk of bias found no significant difference in vomiting between somatostatin analog and placebo in their primary end points [34, 37]. A secondary analysis of a randomized control trial originally published in 2015 examined the health-related quality of life in patients with inoperable MBO and found no difference in quality-of-life scores [38].

#### Thienobenzodiazepene (second-generation) antipsychotic

*Suggestion: Thienobenzodiazepene antipsychotic (e.g., olanzapine) may reduce nausea and vomiting in MBO (level of evidence: IV; grade: D).*

Olanzapine is a second-generation thienobenzodiazepine antipsychotic that antagonizes serotonin 5-HT_3_ and 5-HT_2c_ and dopamine D_2_ receptors, which may be responsible for its anti-emetic effects. We identified one cross-sectional study, published in 2012, that found olanzapine reduced the average nausea scores and frequency of vomiting in patients with advanced cancer and partial BO [39].

#### Laxatives

*Suggestion: Oral osmotic laxatives should be considered in the management of impaired bowel movements in partial bowel obstruction but should be avoided in complete MBO (level of evidence: V; grade: D).*

Patients with MBO present with reduced or no bowel movements. The review did not identify any studies that specifically examined the use of laxatives in this context. If patients have complete MBO, the use of laxatives is not recommended. Whereas, if patients have partial BO, the cautious use of oral osmotic laxatives (e.g., polyethylene glycol 3350, also known as macrogol) can be used. Osmotic laxatives draw water into the lumen of the bowel to soften stool and stimulate peristalsis [40]. The use of bulk-forming laxatives (e.g., psyllium) is not advised as they will increase stool consistency and potentially worsen BO [40]. If a digital rectal examination identifies a full rectum or fecal impaction, suppositories and fecal disimpaction can be considered in partial MBO [41]. However, enemas should be used with caution and are generally not recommended in MBO due to the risk of bowel perforation. Stool softeners (e.g., docusate) may be used, but their effect on bowel movement frequency is not well established.

### Analgesics

Pain is experienced by 70 to 90% of patients with MBO [42]. Causes include abdominal distention, bowel spasms, and, in some cases, perforation [42]. Pain can be intermittent, cramping, or continuous in nature [4]. The WHO Guidelines for the Pharmacologic and Radiotherapeutic Management of Cancer Pain in Adults and Adolescents recommend that analgesics should be given by mouth whenever possible [43]. However, patients with MBO often have significant nausea and vomiting and malfunctioning gastrointestinal tracts that prevent ingestion and absorption of oral analgesics. Consequently, the parenteral (subcutaneous or intravenous) and/or transdermal routes of administration should be considered for this population to deliver effective analgesia. A comparison of subcutaneous and intravenous routes found no differences, confirming both routes as feasible, effective, and safe [44].

#### Opioids

*Suggestion: Opioids are commonly used to treat pain associated with MBO, but there is no evidence to support their use (level of evidence: V; grade D).*

Opioids are the mainstay analgesic for the management of moderate and severe cancer-related pain, including in the context of MBO. The review did not identify any studies that specifically examined the use of opioids for pain management in MBO. Further study is warranted, especially given that opioids impair gastrointestinal motility and can cause nausea, vomiting, and constipation [45].

#### Anticholinergics

*Suggestion: The benefit of anticholinergics (hyoscine butylbromide) may be effective to reduce abdominal pain in MBO (level of evidence: III; grade: D).*

Hyoscine (scopolamine) butylbromide is an anticholinergic agent that is widely used to treat spasmodic abdominal pain. In addition to reducing gastrointestinal secretions, it slows propulsive peristalsis and relaxes smooth muscles of the gut. Hyoscine butylbromide is commonly recommended for the management of the inoperable MBO but scarce evidence supports its use. A 2016 systematic review [17] identified two randomized clinical trials with high Cochrane risk of bias that found somatostatin analog (e.g., octreotide) were more effective than hyoscine butylbromide in reducing continuous pain [20, 21]. but one trial did not report this finding [22]. Whereas, all three trials did not find a significant difference in colicky pain between octreotide and hyscine butylbromide [20–22]. Two case reports reported hyoscine butylbromide in combination with other drugs (e.g., morphine, octreotide, and dexamethasone) was effective in reducing abdominal pain [22, 23].

#### Corticosteroids

*Suggestion: The use of steroids may help with the acute symptoms of MBO and can be used for short-term benefit (level of evidence: III; grade: B).*

The role of corticosteroids in MBO is complex, and the mechanism of action is not completely understood. Corticosteroids likely have an anti-inflammatory and anti-secretory effect, which may help decrease intestinal wall edema, promote salt and water absorption in MBO and, therefore, help with acute management of pain, nausea, and vomiting [46]. Dexamethasone is generally the preferred corticosteroid given its potent anti-inflammatory effect and lack of sodium-retaining properties [47]. The optimal dose of corticosteroids for MBO management is not well established. A dose between 4 and 16 mg of dexamethasone daily may be considered [34, 48]. In cases of no symptomatic improvement in 3 to 5 days, discontinuation should be considered [48].

Unselected and uncontrolled case series have reported a benefit of corticosteroids for the management of BO, yet it is challenging to attribute whether the resolution of MBO related symptoms is due to the medication or potential spontaneous resolution [30, 49, 50]. Moreover, some of these reports look at combination strategies of corticosteroids with other therapies, which makes it challenging to attribute the resolution of MBO to the corticosteroid therapy per se [34]. A retrospective cohort study retrieved information from a Japanese national medical claims database that included 3,090 adult patients with MBO treated with octreotide [51]. Octreotide alone, in combination with H_2_ antagonists, proton pump inhibitor, or corticosteroids, was administered in 53%, 14%, 11%, and 12% of patients, respectively. A secondary endpoint of the study was the assessment of nasogastric tube removal at 4 days. Of the 1595 patients who underwent a nasogastric tube insertion, those receiving corticosteroids with octreotide had a higher odds ratio (OR) of nasogastric tube removal within four days of insertion compared to those who did not receive corticosteroids (OR 1.16; 95% confidence interval [CI] 1.08–1.23).

A Cochrane systematic review and meta-analysis (updated search performed in 2017, with stable review) assessed the role of corticosteroids for MBO in patients with advanced gynecologic or gastrointestinal tumors [48]. Three double-blind placebo-controlled clinical trials involving 89 patients were included in the analysis. Two of the studies included in the meta-analysis were unpublished. In two of the trials, patients with a history of gastrointestinal hemorrhage, active peptic ulceration, or signs of peritonitis were excluded, and intravenous dexamethasone at 16 mg for 5 days was used [48, 52]. The third trial used methylprednisolone 40 and 240 mg intravenously for 3 days (dexamethasone equivalent 8 and 48 mg; dosage was not considered in the analysis) [48]. The primary outcome of the study was the resolution of BO within 10 days of symptom onset. There was a trend that corticosteroids may be beneficial, with a point estimate of 0.51 (95% CI 0.19–1.43). The number needed to treat with corticosteroids to resolve a BO was six. There was no effect of corticosteroids on mortality at one month. The study also concluded that the incidence of adverse events related to corticosteroids is very low; however, the use of the lowest effective dose for the shortest period should be considered to avoid long-term toxicity [48]. Concerns of long-term use include oral candidiasis, muscle proximal weakness, cushingoid habitus, gastric ulceration, infection risk, mood swings, and sleep disturbances, among others.

In a multi-center randomized trial assessing the role of octreotide in control of vomiting, dexamethasone (8 mg daily, intravenously), along with ranitidine and parenteral hydration, was considered the standardized supportive therapy for acute management of MBO [34]. There were no significant differences in the number of days free of vomiting between the octreotide and placebo groups. However, there was a significant drop in the mean number of vomiting episodes in both groups, suggesting that the standardized supportive therapy was helpful. The relative contribution of dexamethasone and/or ranitidine in the reduction of vomiting could not be addressed given the study design. No other randomized controlled trials assessing the role of corticosteroids have been detected in our review following the updated search of the Cochrane systematic review.

#### Oral water-soluble contrasts

*No guideline possible. There are insufficient data to determine the efficacy of oral water-soluble contrasts in MBO (level of evidence: V; grade D).*

Oral water-soluble contrasts are iodinated contrasts that are opaque on plain x-rays [53]. The most common form used is gastrografin, a hyperosmolar solution that is a combination of sodium diatrizoate and meglumine diatrizoate. They can be used in the management of adhesive BO as a tool to predict the resolution of adhesive small BO with conservative management, and it may also decrease the need for surgery and hospital stay [53]. A Cochrane systematic review assessed the role of oral water-soluble contrast in MBO [53] and identified only one randomized double-blinded placebo-controlled feasibility study with a high risk of bias that enrolled nine patients [54]. The study assessed the role of gastrografin compared to placebo in patients with MBO and no indication for surgery or endoscopic interventions. The rate of resolution of MBO or ability to predict resolution of MBO was not reported. No safety signals were detected. A retrospective study assessed the role of the gastrografin in the management of BO [55]. The administration of gastrografin challenge was considered safe. In the subgroup analysis of patients with active malignancy (*n* = 63), there were no differences in the need for surgical exploration or length of hospital stay between those who did and did not receive gastrografin. No other reports have been detected in our search following the publication of the Cochrane review.

### Bowel decompression

Nasogastric tube and percutaneous gastrostomy insertion, either endoscopically or radiologically guided, are established techniques to provide enteral feeding. In the case of MBO, these measures are used as a venting/decompression procedure. The following sections provide suggestions/recommendations for all bowel decompression interventions and their associated existing evidence:

#### Nasogastric tube

*Suggestion: Nasogastric tube may be used for temporary decompression in acute MBO (level of evidence: V; grade: D).*

Temporary decompression via a wide bore nasogastric tube can evacuate a large amount of pooled gastric secretions, particularly during acute MBO episodes, and may improve symptoms [56]. Based on clinical expertise and existing guidelines, it is not suggested for chronic MBO management because the nasogastric tubes are not well tolerated when placed for a prolonged time [57, 58]. Nasogastric tubes can become occluded or displaced, which may require flushing or replacement. Other potential complications include nasal cartilage erosion, otitis media, aspiration pneumonia, esophagitis, and bleeding [59].

#### Endoscopic or percutaneous gastrostomy tube

*Suggestion: Endoscopic or percutaneous gastrostomy tube may be used for gastric decompression in MBO (level of evidence: IV; grade B evidence).*

A more permanent decompression of gastric contents can be achieved through the placement of an endoscopic or percutaneous gastrostomy tube, also known as “venting gastrostomy”. This review identified 29 studies that suggest gastrostomy insertion is generally feasible [60–87], with a high reduction in symptoms of nausea and vomiting. Major complications were rare, with most complications classified as minor wound infections or leakage of fluid around the tube. The presence of ascites is not an absolute contraindication to the insertion of percutaneous venting gastrostomy in patients with MBO [61, 68, 78]; however, it is reasonable to suggest that ascitic drainage with paracentesis or placement of an intraperitoneal catheter is performed to reduce potential complications.

#### Percutaneous transesophageal gastro-tubing

*Suggestion: Percutaneous transesophageal gastro-tubing may be used for gastric decompression in MBO (level of evidence: IV; grade: C).*

The placement of percutaneous transesophageal gastro-tubing (PTEG) may be used to decompress gastric contents. This review identified four studies with a limited number of patients with MBO that are not candidates for surgical decompression or percutaneous/endoscopic gastrostomy tubes [88–91]. A Japanese randomized controlled trial assessed PTEG or nasogastric (NG) tube in 40 patients with inoperable MBO. The PTEG resulted in fewer symptoms and higher quality of life compared with NG tube [88]. The primary endpoint was symptom palliation for two weeks (non-validated questionnaire), and showed lower symptom burden in those patients with PTEG. Secondary endpoints included quality of life measures (EQ-5D and SF-8 scores), which were significantly higher in the PTEG group. No differences in overall survival were detected between the two groups.

#### Palliative surgery and stent

*Recommendation and suggestions*

- *Self-expanding metallic stents are the preferred alternative for the management of single-level large bowel obstruction when technically feasible and in the absence of colonic perforation (level of evidence: II; grade: B). Surgical palliation may be considered an alternative in selected cases.*
- *In the case of a multi-level obstruction, palliative surgical intervention may be considered in a highly selected population (level of evidence: IV; grade: B).*
- *Patients with advanced cancer that undergo palliative surgery for MBO are at high risk of surgical complications, and less invasive surgical interventions should be considered (level of evidence: IV; grade: B).*

Interventions in single-level obstruction may include decompression methods or open surgery with bypass or stoma [92]. Obtaining a surgical opinion should be considered at the time of MBO diagnosis. Stoma formation is preferred if the length of the proximal intestine ensures gastrointestinal autonomy (low risk of short bowel syndrome) or if the risk of complications related to intra-abdominal anastomosis is too high [93]. The large bowel decompression options may include colonic decompression tubes, ablative methods, and the use of self-expanding metal stents (SEMS) [94]. The SEMS are generally the preferred decompression method as they have shown higher success rates than decompression tubes [95, 96]. Decompression methods are not indicated in patients with a bowel perforation [94].

Most studies assessing the role of SEMS in MBO have included mixed populations (curative intent with the stent placement as a bridge-to-surgery and palliative) [97–102]. The role of the stent as bridge-to-surgery is out of the scope of this guideline. In a meta-analysis of patients with large BO caused by colorectal cancer undergoing SEMS placement or emergency surgery with palliative intent, eighteen studies (randomized controlled trials and comparative observational studies) with 1518 patients were included. Results showed that 30-day mortality was higher in those patients undergoing a surgical procedure (OR 0.4; 95% CI 0.28–0.69) [103]. Early complications were more frequent in the surgical group, whereas late complications occurred more frequently after SEMS, mainly re-obstructions. Another meta-analysis in the same setting only including randomized controlled trials (four studies, 125 patients), showed no differences in 30-day mortality or mean survival between those patients undergoing SEMS or emergency surgery, with a shorter hospital stay in the SEMS group [104]. Another randomized controlled trial assessed the role of stent insertion or surgical decompression for non-curable large BO [105]. Results showed that the combined costs were lower in those patients treated with a stent. In terms of quality of life, measured by European quality of life five dimension (EQ-5D) scores, no differences were detected at 4-weeks between the two groups.

Most of the studies assessing the role of palliative surgery for multi-focal or small bowel MBO are retrospective in nature. A Cochrane systematic review of studies that assessed the role of surgery for MBO in advanced gynecologic or colorectal cancer identified 43 studies with a total of 4265 participants [2]. Most studies were retrospective, of low methodological quality and high risk of bias. Additionally, some studies included mixed populations with the benign and malignant causes of BO. Re-obstruction rates post-surgical laparotomy, when reported, ranged from 10 to 63%, with limited time to re-obstruction data. The ability to feed orally ranged between 30 and 100%. When reported, postoperative mortality within 30 days was 0 to 32%, and postoperative morbidity ranged from 22 to 87%. It was not possible to conclude whether there was the benefit of using surgery in this setting. Following the publication of the Cochrane review, no randomized trials have been detected in our review. However, other systematic reviews have focused on studies assessing the role of surgery in specific populations with MBO, including patients with small BO or peritoneal carcinomatosis [93, 106]. Similarly, it was not possible to conclude if there is a benefit on surgery in this setting, as most studies were retrospective, of low methodological quality, and high selection bias in those deemed appropriate for surgical intervention. It may be reasonable to select patients that are more likely to benefit from the procedure, including good performance status, and to focus the surgical intervention on relieving symptoms.

### Nutrition

Oral intake is significantly impaired in MBO and raises important questions about hydration and nutrition, which should be carefully considered and discussed with the patient and their substitute decision-makers.

#### Diet

*Suggestions:*

- *When a patient is initially diagnosed with MBO, they should be made Nil Per Os (NPO; nothing by mouth), and then when the acute MBO resolves fully or partially, a symptom led, slow and graded reintroduction to oral diet is recommended. This may include clear fluids, free or full fluids, texture modified low fiber diet (soft, minced, and pureed), and if tolerated, back to normal textured low fiber diet (level of evidence: IV; grade: B).*
- *Nutrition interventions should be initiated in patients with advanced cancers only where the benefits of these interventions on quality of life and survival outweigh the risks, with clear expectations discussed by a multidisciplinary team with patients and families (level of evidence: IV; grade: B).*

Patients admitted with acute onset of an MBO are made nil by mouth with the addition of a decompressive procedures to control symptoms such as nausea and vomiting [2, 107, 108]. Managing the nutrition requirements of patients presenting with advanced cancers and MBO is controversial and ethically challenging as there is no consensus due to scant published evidence [5, 109, 110]. Nutrition interventions must be considered in the context of the patient’s prognosis with the focus on prioritising the patient’s wishes [111]. Awareness of the sociocultural meaning of food and nutrition for many people is an important consideration and may contribute to increases in quality of life [111, 112].

As a patient’s acute MBO resolves, either fully or partially, a symptom led, slow, and a graded reintroduction to oral diet is recommended. This may include clear fluids, free or full fluids, texture modified low fiber diet (soft, minced, and pureed) and back to normal textured low fiber diet. A low fiber diet is one that contains a maximum of 10 g of fiber per day. A low fiber diet is thought to be beneficial due to a reduction in stool bulk which may lead to reduced pain, abdominal cramps, gas or feeling of fullness, particularly in those people with ongoing subacute BO [110]. General recommendations also include a grazing eating pattern, with small volumes of food and fluid consumed at any one time. Nutrition education to assist the patient and family to modify their diet according to symptoms is recommended to enhance autonomy and self-management.

##### Hydration

*Suggestions*

- *Parenteral hydration does not prevent or improve symptoms, such as thirst or dry mouth, nor does it increase survival, and in excessive amounts, it may bring on fluid overload, peripheral and pulmonary edema (level of evidence: III; grade: B).*
- *Parenteral hydration should not be initiated routinely in the last days of life (level of evidence: III; grade: B).*

Hydration is an element of parenteral nutrition,^42^ yet hydration may be delivered without nutrition, and it should be considered separately as a means of palliative care to relieve symptoms, not prolonging life [113]. Parenteral hydration (PH) means providing fluids infusion by the route other than oral or enteral (i.e., intravenously or subcutaneously; hypodermoclysis) [111]. Hypodermoclysis is an effective and safe route of hydrating a patient up to 1500 mL/day, with few local adverse effects [114]; with attention to water and electrolytes balance. PH is an element of palliative care and should follow predefined realistic treatment goals.

In a small prospective randomized trial of patients with inoperable MBO, the amount of parenterally supplied fluids was not associated with thirst, dry mouth intensity, or abdominal distention, but a volume of >500 mL/day might reduce nausea and drowsiness [20]. However, a high level of PH may result in more gastrointestinal secretions [3].

A Cochrane review found an insufficient number of low risk of bias studies, which does not allow the formulation of any clinical practice recommendations about medically assisted PH in palliative care patients [115]. All the studies included in the Cochrane review only had participants with advanced cancer, but it was not reported whether they had MBO. Our review did not detect specific studies assessing PH at the end of life, specifically in patients with MBO. At the end of life, PH may not be beneficial [116]. In a systematic review of practices in the last week of the life of patients with cancer, PH did not improve symptoms such as thirst and delirium, although data to support or discourage it is scarce [117]. Dehydration is a frequent cause of delirium, and PH may appear effective, except in the dying phase when even moderate amounts of artificial hydration may be harmful. PH in the dying phase may increase the risk of fluid overload, peripheral swelling, ascites, and pulmonary edema [116]. In a multi-center, double-blind placebo-controlled randomized study, subcutaneous infusion of 1000 mL daily did not improve symptoms of dehydration (fatigue, myoclonus, sedation, and hallucinations), quality of life, or survival at the end of life [118].

##### Parenteral nutrition

*Suggestions:*

- *HPN may be beneficial and maintain the quality of life in a very selected group of patients with MBO (level of evidence: IV; grade: D).*
- *Central venous access is preferred for HPN delivery (level III, grade: B).*
- *In end-of-life HPN, should be discontinued (or not initiated) as it raises the risk of complications and may prolong suffering (level of evidence: V; grade: D).*

The following terms are used to describe parenteral nutrition: “total parenteral nutrition” (TPN): nutrition administered exclusively intravenously; “supplemental parenteral nutrition (SPN)”: nutrition administered intravenously and enterally, regardless of proportion, orally or by artificial access; and “home parenteral nutrition” (HPN): parenteral nutrition (regardless TPN or SPN) administered at home.

Feeding through the peripheral veins may cause phlebitis and the need for the reintroduction of vein access. It may cause patient suffering and require additional medical procedures. Feeding through the central veins (e.g., Broviac catheter, vascular access port, peripherally inserted central catheter) is advised, as it ensures long-term safe access upon the strict aseptic protocol is followed [119, 120].

Most studies assessing the role of the parenteral nutrition are retrospective. A Cochrane systematic review assessed the effectiveness of HPN in survival and quality of life in patients with inoperable MBO [121]. Thirteen studies (all observational with a high risk of bias), including 721 participants were included. The median survival intervals were variable, between 15 to 155 days [121]. Only three studies reported validated quality of life measures, showing equivocal results (one study reported improvements during the first three months and two studies showed an equal number of patients with improved and deteriorated quality of life) [122–124].

HPN can be associated with the risk of complications (metabolic and/or catheter-related). In the Cochrane systematic review, adverse events were measured in nine studies, showing that between 6 and 21% of patients developed a central venous catheter infection or were hospitalized because of complications related to parenteral nutrition [121]. The use of HPN also involves financial, personnel, and infrastructure resources. A meta-analysis included a health economic evaluation of HPN in this setting [125].

There is a need to weigh the benefits and risks when recommending HPN, including the predicted cancer-related survival [125–127]. Potential prognostic criteria for survival and benefit of HPN may include (i) histopathological type of the tumor—slow-growing and chemo-sensitive cancer [128–131], (ii) performance status—ECOG <2, (iii) no fluid retention (peripheral edema, pleural or peritoneal effusion), (iv) no anemia, and (v) no hypoalbuminemia [132]. This evaluation should be reassessed over time.

Most patients may not benefit from HPN due to clinical deterioration resulting from cancer progression, chronic cancer treatment [133], latent infections [134], depression (up to 24%) [135, 136], and suboptimal nutritional treatment [137]. Malnutrition and cachexia in patients with advanced cancer and MBO are usually irreversible and treatment-resistant [138]. Therefore, the goal of the treatment is to maintain the nutritional status and achieve functional (through physiotherapy and nutrition), symptomatic, associative and mental function improvement.

The European Society for Clinical Nutrition and Metabolism (ESPEN) practical guideline for clinical nutrition in patients with cancer suggests that in dying patients, treatment recommendations should be based on comfort [109]. Parenteral hydration and nutrition are unlikely to provide any benefit for most patients at the end of life [109].

### Psychosocial support

The course of MBO can be unpredictable with a range of complex and challenging symptoms, which can be difficult to manage for both the patient and their family. The importance of family and supportive care is firmly established within palliative and end-of-life care literature [139, 140]. Although the need for emotional and psychological support for both patient and family in the context of MBO is equally acknowledged, high-quality studies which focus specifically on the context of MBO are limited [141, 142]. Evidence has been based on clinical case reviews, small scale qualitative descriptive studies or current best practice opinions.

The unmet psychosocial need has been identified as a significant issue in patients with ovarian cancer and MBO, as it is often poorly addressed. The need for a multidisciplinary approach to care is promoted [143]. A qualitative study described the benefits of a model of supported self-management for women with advanced gynecological cancers and MBO attending out-patient clinics. Clear communication, counselling and referral to early palliative care were identified as important considerations [144]. A separate qualitative study with oncologists treating gynecological cancers identified that early palliative care referral was beneficial, particularly in situations where patients were not candidates for surgery [145]. However, patients’ expectations of their oncologists were not always met, suggesting strengthening of communication strategies and protocols is needed.

Given the nature and range of complex symptoms associated with MBO, early palliative care has been suggested. Optimal treatment requires a realistic assessment of goals of care with important communication about prognosis, management of symptoms, and end-of-life care. A recent review of the surgical management of MBO suggests careful decision-making with patient and family to ensure the most appropriate outcome [146]. A 2018 systematic review focused on the burden of care placed on family caregivers as a result of home-based parenteral nutrition for women with advanced ovarian cancer and MBO found that caregivers described an experience of vulnerability and family disruption caused by the decision to care for the patient at home. Conversely, in acknowledging the challenges placed on the family, patients recognized the lifeline given to them in terms of spending quality time with their family by this option for home care [121].

## Conclusion

MBO is a significant complication for patients living with cancer. The approach should be multi and inter-disciplinary to improve the management of these patients and support their families. Studies indicate the need to improve the quality of research and subsequent interventions in this domain (refer to Table 3 for a summary of suggestions and recommendations). Communication around changing goals of care is essential to foster clear and decisive clinical decision making in partnership with patients and families. Further prospective and innovative studies are needed to improve the care for patients with MBO.

## Supplementary Information

Below is the link to the electronic supplementary material.

Supplementary file1 (DOCX 34 KB)

## Data Availability

The review search strategy and data are available upon reasonable request.
