# Supplementary material for: MASCC multidisciplinary evidence-based recommendations for the management of malignant bowel obstruction in advanced cancer
Source: Support Care Cancer. 2022 Mar 10;30(6):4711–28. doi: 10.1007/s00520-022-06889-8 (PMC9046338; doi:10.1007/s00520-022-06889-8)
Supplement: Supplementary file 1 — Supplementary file1 (DOCX 34 KB) [file 520_2022_6889_MOESM1_ESM.docx]

APPENDIX

1. Medline search strategy.

**Ovid MEDLINE(R) ALL 1946 to March 23, 2021**

|  |  |  |  |  |  |  |
| --- | --- | --- | --- | --- | --- | --- |
| **#** | **Searches** | **Results** | **Type** |  |  |  |
|  | | | | | | |
| 1 | exp Intestinal Obstruction/ | 47698 | Advanced |  |  |  |
| 2 | (bowel? adj3 obstruct*).mp,kw. | 12236 | Advanced |  |  |  |
| 3 | (gastro* adj3 obstruct*).mp,kw. | 2008 | Advanced |  |  |  |
| 4 | (intestin* adj3 obstruct*).mp,kw. | 38651 | Advanced |  |  |  |
| 5 | (duoden* adj3 obstruct*).mp,kw. | 4498 | Advanced |  |  |  |
| 6 | (ile* adj3 obstruct*).mp,kw. | 1174 | Advanced |  |  |  |
| 7 | (jejun* adj3 obstruct*).mp,kw. | 375 | Advanced |  |  |  |
| 8 | (meckel* adj3 obstruct*).mp,kw. | 107 | Advanced |  |  |  |
| 9 | ((colon* or colic*) adj3 obstruct*).mp,kw. | 3237 | Advanced |  |  |  |
| 10 | ((c?ecum* or c?ecal*) adj3 obstruct*).mp,kw. | 123 | Advanced |  |  |  |
| 11 | (sigmoid* adj3 obstruct*).mp,kw. | 275 | Advanced |  |  |  |
| 12 | (rect* adj3 obstruct*).mp,kw. | 621 | Advanced |  |  |  |
| 13 | or/1-12 | 62503 | Advanced |  |  |  |
| 14 | exp Neoplasms/ | 3433371 | Advanced |  |  |  |
| 15 | neoplas*.mp,kw. | 2994798 | Advanced |  |  |  |
| 16 | cancer*.mp,kw. | 1920433 | Advanced |  |  |  |
| 17 | tumo?r*.mp,kw. | 2210610 | Advanced |  |  |  |
| 18 | malig*.mp,kw. | 610726 | Advanced |  |  |  |
| 19 | metast*.mp,kw. | 604719 | Advanced |  |  |  |
| 20 | micrometast*.mp,kw. | 6978 | Advanced |  |  |  |
| 21 | macrometast*.mp,kw. | 978 | Advanced |  |  |  |
| 22 | oligo*.mp,kw. | 437343 | Advanced |  |  |  |
| 23 | or/14-22 | 5011949 | Advanced |  |  |  |
| 24 | "Quality of Life"/ | 206160 | Advanced |  |  |  |
| 25 | Quality-Adjusted Life Years/ | 13026 | Advanced |  |  |  |
| 26 | exp "Outcome Assessment (Health Care)"/ | 1178876 | Advanced |  |  |  |
| 27 | exp Patient Outcome Assessment/ | 13492 | Advanced |  |  |  |
| 28 | Patient Reported Outcome Measures/ | 7761 | Advanced |  |  |  |
| 29 | Treatment Outcome/ | 1013800 | Advanced |  |  |  |
| 30 | Treatment Failure/ | 35801 | Advanced |  |  |  |
| 31 | Symptom Assessment/ | 5804 | Advanced |  |  |  |
| 32 | Disease Management/ | 38734 | Advanced |  |  |  |
| 33 | Patient Safety/ | 22085 | Advanced |  |  |  |
| 34 | exp Safety Management/ | 20567 | Advanced |  |  |  |
| 35 | Survivors/ | 25192 | Advanced |  |  |  |
| 36 | Cancer Survivors/ | 4786 | Advanced |  |  |  |
| 37 | Survival/ | 4804 | Advanced |  |  |  |
| 38 | Survival Rate/ | 179056 | Advanced |  |  |  |
| 39 | exp Survival Analysis/ | 308214 | Advanced |  |  |  |
| 40 | Disease-Free Survival/ | 76483 | Advanced |  |  |  |
| 41 | Disease Progression/ | 171597 | Advanced |  |  |  |
| 42 | (qualit* adj4 life).mp,kw. | 376723 | Advanced |  |  |  |
| 43 | (life adj3 year? adj3 adjusted).mp,kw. | 22151 | Advanced |  |  |  |
| 44 | QOL?.mp,kw. | 41879 | Advanced |  |  |  |
| 45 | QALY?.mp,kw. | 11417 | Advanced |  |  |  |
| 46 | HRQOL?.mp,kw. | 17940 | Advanced |  |  |  |
| 47 | (outcome? adj3 assess*).mp,kw. | 170995 | Advanced |  |  |  |
| 48 | (patient? adj3 outcome?).mp,kw. | 280095 | Advanced |  |  |  |
| 49 | (patient? adj3 report*).mp,kw. | 225291 | Advanced |  |  |  |
| 50 | PRO?.mp,kw. | 270265 | Advanced |  |  |  |
| 51 | PROM?.mp,kw. | 7931 | Advanced |  |  |  |
| 52 | PROMIS?.mp,kw. | 105467 | Advanced |  |  |  |
| 53 | (treatment? adj3 outcome?).mp,kw. | 1087283 | Advanced |  |  |  |
| 54 | (treatment? adj3 failur*).mp,kw. | 75394 | Advanced |  |  |  |
| 55 | (symptom? adj3 assess*).mp,kw. | 33173 | Advanced |  |  |  |
| 56 | (symptom? adj3 manag*).mp,kw. | 14484 | Advanced |  |  |  |
| 57 | (disease? adj3 manag*).mp,kw. | 87769 | Advanced |  |  |  |
| 58 | (patient? adj3 manag*).mp,kw. | 175570 | Advanced |  |  |  |
| 59 | (patient? adj3 safe*).mp,kw. | 78688 | Advanced |  |  |  |
| 60 | surviv*.mp,kw. | 1502406 | Advanced |  |  |  |
| 61 | ((recur* or re-cur*) adj3 free*).mp,kw. | 23984 | Advanced |  |  |  |
| 62 | ((reoccur* or re-occur*) adj3 free*).mp,kw. | 9 | Advanced |  |  |  |
| 63 | (relaps* adj3 free*).mp,kw. | 13069 | Advanced |  |  |  |
| 64 | (residual* adj3 free*).mp,kw. | 1108 | Advanced |  |  |  |
| 65 | (disease? adj3 free*).mp,kw. | 134580 | Advanced |  |  |  |
| 66 | (disease? adj3 progres*).mp,kw. | 307035 | Advanced |  |  |  |
| 67 | (disease? adj3 exacerbat*).mp,kw. | 8462 | Advanced |  |  |  |
| 68 | (respon* adj3 evaluat* adj3 (tumo?r* or cancer*)).mp,kw. | 2127 | Advanced |  |  |  |
| 69 | recist?.mp,kw. | 4732 | Advanced |  |  |  |
| 70 | mrecist?.mp,kw. | 454 | Advanced |  |  |  |
| 71 | resolution?.mp,kw. | 425721 | Advanced |  |  |  |
| 72 | or/24-71 | 4301708 | Advanced |  |  |  |
| 73 | 13 and 23 and 72 | 5190 | Advanced |  |  |  |
| 74 | (exp animals/ or exp animal experiment/) not humans/ | 4804408 | Advanced |  |  |  |
| 75 | 73 not 74 | 5137 | Advanced |  |  |  |
| 76 | limit 75 to "all child (0 to 18 years)" | 560 | Advanced |  |  |  |
| 77 | limit 75 to "all adult (19 plus years)" | 3721 | Advanced |  |  |  |
| 78 | 76 not 77 | 231 | Advanced |  |  |  |
| 79 | 75 not 78 | 4906 | Advanced |  |  |  |
|  | | |  |  |  |  |

**Appendix B**: Summary of evidence about anti-emetics used for malignant bowel obstruction management.

| **Author (Year)** | **Study Design** | **Study Population** | **Intervention** | **Comparator** | **Outcomes** |
| --- | --- | --- | --- | --- | --- |
| Berger (2016) | Case series | Bowel dysfunction (n=7), BO (n=12) | Metoclopramide, octreotide, dexamethasone |  | 11/12 patients reported improved nausea by treatment day 1. 7/12 patients had moderate-severe pain on presentation and all 7 reported tolerable pain by treatment day 1. Median time to resumption of oral intake was 2 days in 8 evaluable patients. |
| Baines (1985) | Cross-sectional study | BO | Prochlorperazine, chlorpromazine, methotrimeprazine, haloperidol, metoclopramide, domperidone |  | Prochlorperazine, chlorpromazine, haloperidol, methotrimeprazine were effective for vomiting. Domperidone and metoclopramide were ineffective for vomiting. |
| Clare (2002) | Case series | BO | Octreotide, lanreotide |  | Transition from octreotide subcutaneous infusion to intermittent lanreotide injections was ineffective for vomiting |
| Currow (2015) | Randomized control trial | BO with vomiting, age ≥ years | Octreotide | Placebo (normal saline) | Patients in both intervention and placebo groups received dexamethasone, ranitidine, hydration and as needed parenteral opioids, hyoscine butylbromide and haloperidol.  No statistical differences in number of vomiting between the two group (p=0.71), total number of people free of vomiting for 72 hours (p=0.67) and mean number of days free of vomiting in each group (p=0.47). |
| Davis (1999) | Case report | BO | Glycopyrrolate, haloperidol, hydromorphone |  | Combination of glycopyrrolate, haloperidol and hydromorphone were effective for nausea and vomiting. |
| De Conno (1991) | Case series | Ovarian Ca, BO | Hyoscine butylbromide and octreotide |  | Hyoscine butylbromide and octreotide are effective for symptom management of refractory MBO. |
| Fainsinger (1994) | Cross-sectional study | BO: complete (n=10), incomplete (n=5) | Metoclopramide, dimenhydrinate, haloperidol, hyoscine butylbromide, cisapride, domperidone, chlorpromazine |  | Medications used for nausea/vomiting: 12/15 metoclopramide, 7/15, dimenhydrinate. 3/15 haloperidol, 2/15 patients hyoscine butylbromide, 2/15 domperidone, 1/15 cisapride, 1/15 chlorpromazine |
| Hisanaga (2010) | Cross-sectional study | ≥ 20 years, MBO: based on imaging and symptoms, inoperable | Octreotide,  As needed haloperidol, prochlorperazine, morphine, fentanyl |  | Abdominal distention (p=0.001), anorexia (p=0.001), nausea (p<.001), vomiting (p<.001), fatigue (p<.001) and thirst (p<.001) were significantly improved on day 4 compared with baseline. Nausea improved in 72% of the efficacy analysis set and duration of nausea was decreased in 52%. Quality of life improved in 56%. Mean number of daily vomiting episodes decreased significantly on day 4 (P = .001). |
| Hwang (2013) | Cross-sectional study | BO - inoperable | Octreotide |  | No statistical difference between patients with BO prescribed octreotide by the medical vs surgical services in the following factors: inpatient day octreotide was initiated, patient’s daily octreotide dose, patient’s cumulative octreotide dose, days receiving octreotide, length of stay, age, cancer stage, lines of chemotherapy, cancer type, overall survival |
| Kaneisha (2012) | Cross-sectional study | Incomplete BO: upper GI (n=11), lower GI (n=9) | Olanzapine |  | 18/20 had reduction in nausea intensity. Average frequency of vomiting |
| Khoo (1994) | Cross-sectional study | BO, ≤ 2 months life expectancy, Karnofsky score <50%, intractable vomiting (WHO grade 4) that failed anti-emetics, steroids and/or nasogastric drainage for at least 24 hours | Octreotide |  | Response according to WHO emesis scale: 14/24 patients had complete response (WHO 0). 4/24 had partial response (WHO 1-2). Control of vomiting occurred within 2-4h of achieving correct total daily dose. Median initial dose to control vomiting in responding patients was 300mcg/day. |
| Kubota (2013) | Cross-sectional study | Urological cancer, BO | Octreotide |  | Mean time to control vomiting 1.6 days. Improvement in subjective vomiting: partial response 9/14, complete response 4/14. Nasogatric tube discontinued in 4/4 patients |
| Mangili (1996) | Cross-sectional study | Advanced ovarian cancer, BO | Octreotide |  | Octreotide controlled vomiting for 13/13 pts within a mean of 3.03 days. No reported side effects. |
| Mariana (2012) | Randomized control trial | ≥ 18 years old, peritoneal carcinomatosis, obstruction, ≥2 vomits/day or NG tube, inoperable BO, IV steroids ≥ 5 days and IV PPI | Lanreotide | Placebo | Intention to treat analysis (ITT): No statistical difference between the two groups (p =.24).  Supportive per protocol analysis found significant difference between the two groups (p *=* .05). ITT analysis, on the basis of investigators’ assessments, found significant difference between the two groups (p=.05). |
| Massacesi (2006) | Cross-sectional study | BO refractory to methoclopramide, haloperidol, scopolamine butylbromide and analgesics | Octreotide LAR |  | 8 patients had reduction in nasogastric tube secretions. 4 pts without nasogastric tube had reduction in nausea and vomiting. Reported adverse effect was abdominal pain. |
| Matulonis (2005) | Cross-sectional study | Recurrent epithelial ovarian cancer, BO: inoperable, chronic or intermittent | Octreotide, lanreotide (LAR) |  | Response defined as significant reduction or resolution of nausea/vomiting and/or ability to remove nasogastric tube. 3/13 major response to LAR; 2/13 minor response; 4/13 no response. 4/13 progressive symptoms. 3/13 remained on LAR for more than 9 months. No significant toxicities found with octreotide or LAR. |
| McCaffrey (2020) | Randomized control trial | Same as Currow (2015) | Same as Currow (2015) | Same as Currow (2015) | No statistical difference within (p=0.21) or between groups in mean quality of life (p=0.78). |
| Mercadante (1992) | Case series | BO | Octreotide, buprenorphine, haloperidol |  | Octreotide initiation reduced secretions, vomiting and allowed nasogastric tube to be removed |
| Mercadante (1993) | Cross-sectional study | BO | Octreotide |  | 12/14 patients had improvement in vomiting. |
| Mercadante (1998) | Case report | BO | Metoclopromide, ranitidine, haloperidol, scopolamine butylbromide, octreotide |  | Metoclopramide ineffective in restoring intestinal transit. Ranitidine, haloperidol and scopolamine ineffective in controlling vomiting. Scopolamine and octreotide combined were effective in stopping vomiting. |
| Mercadante (2000) | Randomized control trial | BO - inoperable | Octreotide | Hyoscine butylbromide | Both intervention and comparator groups were able to use as needed analgesics and anti-inflammatories.  Octreotide significantly reduced number of vomiting episodes (p=0.03 to 0.008), nausea at different time points (p=0.01). Octreotide compared to hyoscine butylbromide was more effective with reducing number of vomiting episodes (p=0.01 to p=0.004) and nausea (p=0.002 to 0.003). |
| Mercadante (2004) | Cross-sectional study | BO | Amidotrizoato, metoclopramide, octreotide, dexamethasone |  | Bolus of amidotrizoato followed by combination of metoclopramide, octreotide and dexamethasone resolved vomiting and recovered intestinal transit for 14/15 patients. |
| Mystakidou (2002) | Randomized control trial | BO - inoperable | Octreotide | Hyoscine butylbromde | Both groups received chlorpromazine. Octreotide significant decreased nausea (p=0.007), vomiting (p=0.004), fatigue and anorexia compared to patients who received hyoscine butylbromide. |
| Muir (2001) | Case report | BO | Haloperidol, octreotide |  | As needed, haloperidol is recommended for BO. If ineffective, then suggest addition of octreotide. |
| Obita (2016) | Randomized control trial | BO - inoperable | Somatostatin analogues | Placebo and/or other anti-emetic agents | Two adequately powered multicenter RCTs with a low Cochrane risk of bias reported no significant difference between somatostatin analogues and placebo in their primary end points (number of days free of vomiting, total number of people completely free of vomiting for 72 hours, proportion of patients with ≤1 vomiting episode/day, no recurrence of vomiting after NG tube removal for 3 consecutive days). Four RCTs with a high/unclear Cochrane risk of bias reported that somatostatin analogues were more effective than hyoscine butylbromide in reducing vomiting. |
| Peng (2015) | Randomized control trial | Ovarian cancer, BO – inoperable, life expectancy ≥ 2 months | Octreotide | Scopolamine butylbromide | Compared to scopolamine, octreotide significantly reduced GI secretions at 24h, 48h and 72h (p<0.05), number of episodes of vomiting at 24h, 48h and 72h (p<0.05), intensity of nausea at 48h and 72h (0<0.05) and continuous pain at 48h and 72h (p<0.05). |
| Ripamonti (2000) | Randomized control trial | BO - inoperable | Octreotide | Scopolamine | Compared to before treatment, GI secretions were significantly reduced with octreotide at 48 hours (p=.016) and 72 hours (p=0.020) after starting it. Nausea among the home care patients was lower with octreotide compared to scopolamine 48 hours after starting the drugs (p=0.05). |
| Shima (2008) | Cross-sectional study | Hospitalized patients, BO refractory to treatment, ≥2 emesis/day or ≥500mL/day secretions via nasogastric tube | Octreotide |  | 11/25 had resolution or improvement of nausea/vomiting. Potential treatment-related laboratory adverse events occurred in 6/25 patients including thrombocythemia, leukocytosis, increased ALP and increased GGT. |
| Steadman (1996) | Case report | Stage IV pancreatic cancer | Diamorphine, octreotide |  | Octreotide reduced nausea, vomiting |
| Tuca (2009) | Phase II clinical trial | BO | Granisetron, dexamethasone, haloperidol |  | Compared to before treatment, daily granisetron and dexamethasone and as needed haloperidol decrease the severity of nausea (p<0.001), number of vomiting episodes (p<0.001) and abdominal pain (p<0.001). |
| Watari (2012) | Cross-sectional study | Gynecologic cancer, BO, ≥ 1 emesis/day | Octreotide |  | Vomiting completely controlled in 15/22 patients. |
| Weber (2009) | Case series | Advanced gastrointestinal cancers, BO |  | Morphine, buprenorphine, methadone, dexamethasone, prednisone, metoclopramide, ondansetron, octreotide,  hyoscine butylbromide | Analgesics, corticosteroids,  antiemetics, and octreotide were effective to MBO symptoms. |
